# No Difference in Growth Outcomes up to 24 Months of Age by Duration of Exposure to Maternal Antiretroviral Therapy Among Children Who Are HIV-Exposed and Uninfected in Malawi

**DOI:** 10.3389/fped.2022.882468

**Published:** 2022-06-20

**Authors:** Gabriela Toledo, Megan Landes, Monique van Lettow, Beth A. Tippett Barr, Heather Bailey, Claire Thorne, Siobhan Crichton

**Affiliations:** ^1^Great Ormond Street Institute of Child Health, University College London, London, United Kingdom; ^2^Department of Family and Community Medicine, University of Toronto, Toronto, ON, Canada; ^3^Dalla Lana School of Public Health, University of Toronto, Toronto, ON, Canada; ^4^Centers for Disease Control and Prevention, Kisumu, Kenya; ^5^Institute for Global Health, University College London, London, United Kingdom; ^6^Medical Research Council Clinical Trials Unit, University College London, London, United Kingdom

**Keywords:** CHEU, growth, antiretroviral therapy, HIV, growth faltering, children HIV-exposed and uninfected

## Abstract

**Background:**

With the implementation of lifelong antiretroviral therapy (ART) for HIV treatment and prevention, the proportion of children exposed to ART *in utero* from conception is increasing. We estimated the effect of timing of ART exposure on growth of children HIV-exposed and uninfected (CHEU) up to Up to 24 months of age in Malawi.

**Methods:**

Data were collected from a prospective cohort of infants HIV-exposed aged 1–6 months (enrollment) and their mothers with HIV enrolled in the National Evaluation of Malawi’s Prevention of Mother-to-Child Transmission of HIV Programme (2014–2018). Anthropometry was measured at enrollment, visit 1 (approximately 12 months), and visit 2 (approximately 24 months). Weight-for-age (WAZ) and length-for-age (LAZ) were calculated using the WHO Growth Standards. Multivariable mixed-effects models with linear splines for age were used to examine differences in growth by timing of ART exposure (from conception, first/second trimester, or third trimester/postpartum). Models were adjusted for confounders selected *a priori* guided by a conceptual framework. Hypothesized interactions and potential mediators were explored, and interactions with splines were included in final models if *P* < 0.1.

**Results:**

A total of 1,206 singleton CHEU and their mothers were enrolled and 563 completed the follow-up through 24 months of age. Moreover, 48% of CHEU were exposed to ART from conception, 40% from first/second trimester, and 12% from third trimester/postpartum. At enrollment, 12% of infants had low birthweight (LBW), 98% had been breastfed in past 7 days, and 57% were enrolled in an HIV care clinic. CHEU growth trajectories demonstrated cohort-wide growth faltering after the age of 12 months. Of 788 and 780 CHEU contributing to WAZ and LAZ multivariable models, respectively, there was no evidence of differences in mean WAZ or LAZ among those exposed from conception or first/second trimester vs. third trimester/postpartum and no evidence of a difference in WAZ or LAZ rate of change by timing of ART exposure (all interactions *P* > 1.0).

**Conclusion:**

Reassuringly, ART exposure from conception was not associated with decreased WAZ or LAZ in CHEU Up 24 months of age. Overall growth trajectories suggest CHEU experience growth faltering after 12 months of age and may need support through and beyond the first 2 years of life.

## Introduction

With the shift toward maternal lifelong antiretroviral therapy (ART) for HIV treatment and prevention of vertical transmission, an increasing proportion of children born to women living with HIV (WLHIV) are HIV-free and exposed to antiretroviral drugs *in utero* from conception, with prolonged exposure *via* breast milk as well as infant prophylaxis (1). In 2019, of the estimated 15.2 million children HIV-exposed and uninfected (CHEU) worldwide, 78% were exposed to antiretroviral drugs (2). While prevention of vertical transmission programs have improved maternal health and reduced infant HIV acquisition, CHEU remain at higher risk of poor health outcomes than children HIV-unexposed (CHU) from the same settings (3–6).

Child long-term growth and development are strongly influenced by exposures during the first 1,000 days after conception (7, 8). CHEU in resource-limited settings experience complex and multifactorial exposures (9), including maternal ill health, increased risk of infectious disease, poverty, and poor nutrition. Whether *in utero* and early life exposures to HIV and ART have the potential to adversely affect the fetal and postnatal growth independently of other factors is unclear, although there is growing evidence suggesting that this is the case for CHEU (10–17).

Recently, findings from the PROMISE trial in Malawi and Uganda demonstrated that maternal HIV and ART were associated with lower length-for-age z-scores (LAZ) and weight-for-age z-scores (WAZ), and that CHEU were more likely to be stunted at Up to 24 months of age than CHU (15). Evidence on the effect of timing of ART exposure on growth is inconsistent (17–22); some studies have reported poorer growth in CHEU exposed to ART from conception compared with that of later, while others have noted no difference.

Tenofovir disoproxil fumarate (TDF) is a nucleoside reverse transcriptase inhibitor (NRTI), widely used as part of the WHO preferred first-line ART regimen for pregnant and breastfeeding women in Malawi and globally. Findings regarding the effect of *in utero* exposure to TDF and CHEU growth are mixed (19, 23–27). A recent South African study did not observe an association between the duration of TDF exposure *in utero* and early linear growth (23), although others have reported that TDF-based ART exposure was associated with lower LAZ in CHEU at 12 months (27), a higher risk of underweight at the age of 6 months (25), and lower weight and length growth compared with those without TDF exposure (26).

Various studies from high-income and low-income countries have also indicated associations between specific antiretroviral drugs, timing of exposure, and adverse birth outcomes including LBW and preterm birth (28–30). Some findings suggest these adverse birth outcomes are specifically associated with preconception protease-inhibitor-based ART use (31, 32), although findings from other large studies in Botswana (33, 34) and Tanzania (35) indicate that these outcomes may also be linked to nevirapine-based or efavirenz-based ART. Understanding the effects of HIV and duration of ART exposure on long-term growth is challenging, owing to the presence of universal risk factors for poor child health that are prevalent in resource-limited settings with high maternal HIV prevalence. Nevertheless, the implementation of lifelong ART has improved maternal health and addresses other risk factors for poor child growth (1, 36).

Clarifying the role that timing of ART exposure may have on growth is critical for understanding potential origins and pathways underlying CHEU suboptimal growth. From a public health perspective, such evidence is important, given the high prevalence of HIV, child malnutrition, and growth deficiencies in some resource-limited settings. In Malawi, maternal ART coverage is estimated at 98% (37, 38), approximately 7% of the pediatric population is CHEU (2), and 49% of children aged under 5 years experience growth deficiencies (39). The aim of this study was to estimate the association between timing of ART exposure and CHEU growth up to 24 months of age in Malawi. Specifically, we hypothesized that CHEU postnatal growth trajectories vary by duration of exposure to maternal ART.

## Materials and Methods

### Study Design and Participants

Data were collected from a prospective cohort of infants HIV-exposed aged 1–6 months and their mothers living with HIV who were enrolled in the National Evaluation of Malawi’s Prevention of Mother-to-Child Transmission of HIV Programme (NEMAPP) study between October 2014 and May 2016 with follow-up concluded in 2018. The NEMAPP study used a multistage cluster design to randomly sample 54 health facilities across Malawi; the study methodology has been published elsewhere (40). Mothers with a positive rapid HIV test and a positive HIV polymerase chain reaction (PCR) test were invited to participate in the study with their infants. In Malawi, all pregnant and breastfeeding WLHIV were started on lifelong ART (daily fixed-dose tenofovir [TDF] + lamivudine [3TC] + efavirenz [EFV]) at the time of the study.

The NEMAPP study design was for follow-up to 24 months of age, with study visits intended to take place during mothers’ routine ART appointments; visit 1 was at the age of approximately 12 months and visit 2 at the age of approximately 24 months. At each visit, trained study staff interviewed mother–child pairs using questionnaires to collect information on maternal, child, and contextual factors. For a subset of CHEU (long-term follow-up cohort) from 13 of the 54 health facilities, weight, length, and mid-upper arm circumference (MUAC) were measured at enrollment, visit 1, and visit 2, with blood samples collected for HIV ribonucleic acid (RNA) and PCR testing at each visit.

### Inclusion Criteria for Growth Analysis

Singleton children in the long-term follow-up cohort were included if they (i) were confirmed CHEU based on a negative HIV PCR test result at each study visit and (ii) had data on timing of maternal ART initiation available.

### Growth Outcomes

Age- and sex-adjusted WAZ and LAZ scores were calculated using the World Health Organization (WHO) Growth Standards with child age reported in whole months (41). Underweight and stunting were defined as a WAZ and LAZ more than two standard deviations (SD) below the reference median, respectively.

### Primary Exposure, Confounding, and Mediation

The primary exposure was the timing of maternal ART initiation categorized as from conception (mother started ART before pregnancy), first or second trimester (mother started ART ≤ 6 months of pregnancy), and third trimester or postpartum (mother started ART ≥ 7 months of pregnancy or postpartum).

Confounders were selected *a priori* based on a literature review, which identified *maternal characteristics*: maternal age (i.e., <21, 21–34, and 35 + years), parity (i.e., primiparous or multiparous), maternal HIV viral load at study enrollment (i.e., detectable defined as ≥ 40 copies/ml), maternal health at study enrollment (i.e., no illness or sick), maternal undernutrition at study enrollment (i.e., MUAC < 24 cm); *child characteristics*: child sex (i.e., female or male), infant birth weight (in kilograms), age (in whole months), breastfeeding in the past 7 days (i.e., yes or no); and *contextual factors*: maternal employment (i.e., yes or no) and geographical region (i.e., Blantyre, Lilongwe, North and Central, and South Malawi) as confounders.

Other factors explored as potential confounders included antenatal care visits during pregnancy (i.e., 1–2, 3–4, and 5 + visits), maternal education at study enrollment (i.e., yes or no), maternal health at ART initiation (i.e., no illness, a little bit sick, and very sick), maternal timing of HIV diagnosis (i.e., newly diagnosed through the NEMAPP study or already known positive), child receipt of nevirapine prophylaxis (i.e., yes or no), child hepatitis B immunization at study enrollment (i.e., yes or no), child sick visits to health facility or clinic (i.e., 0, 1–2, and 3+ times) in the previous 3 months at study enrollment (or birth for children aged <3 months at enrollment), and child receipt of co-trimoxazole prophylaxis (i.e., yes or no).

We hypothesized that LBW (defined as <2.5 kg) and child morbidity may be on the causal pathway between timing of ART exposure and CHEU growth. Markers for child morbidity included child health at study visit (i.e., well or sick), child history of infectious disease diagnosis (i.e., any diagnosed episode of malaria, diarrhea, pneumonia, meningitis, and/or tuberculosis in the previous 3 months at enrollment or birth for children aged <3 months at enrollment) or since the previous study visit, and child ever hospitalized (i.e., yes or no) since birth. We also hypothesized that (i) child WAZ and LAZ may differ by timing of ART exposure over time and (ii) breastfeeding and undetectable maternal HIV viral load may have a protective effect for WAZ and LAZ potentially through effect modification.

Data were maternal-reported unless noted otherwise; markers for maternal health, prevention of vertical transmission uptake, pregnancy/birth characteristics, and mother–child outcomes were validated using individual medical records from ART Patient Cards, Exposed Infant Patient Cards, and/or Health Passports when possible. Time-updated factors included breastfeeding in the past 7 days, child receipt of co-trimoxazole prophylaxis, child health, child history of infectious disease diagnosis, and child ever hospitalized.

### Statistical Analysis

Descriptive statistics were used to summarize enrollment characteristics and child anthropometry. To identify potential confounders and examine differences between mother–child pairs who were and were not included mixed effects linear regression models, enrollment characteristics were compared by timing of ART exposure and by mother-child pairs who did and did not contribute to WAZ and/or LAZ mixed-effects models. Characteristics at follow-up visits (i.e., breastfeeding in past 7 days, child receipt of co-trimoxazole prophylaxis, child health, child history of infectious disease diagnosis, and child ever hospitalized) among mother–child pairs who were included in WAZ or LAZ mixed-effects models were also described.

**FIGURE 1:**
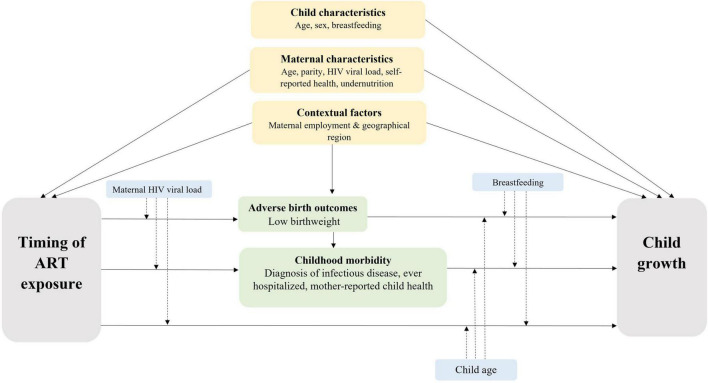
Conceptual Framework. Grey boxes denote primary exposure and outcome; yellow boxes denote *a priori* confounders; green boxes denote potential mediators; dashed arrows from blue boxes denote potential effect modifiers.

Mixed-effects linear regression models were used to examine differences in WAZ or LAZ by timing of ART exposure. The models included linear splines for age with knots fixed at 12 months for WAZ and 13 months for LAZ based on the overall growth trajectory. Random effects were fitted on the child, and a residual exponential correlation structure was applied to account for repeated measures and clustering by the health facility. Multivariable models were adjusted for confounders selected *a priori*, guided by a conceptual framework (Figure 1). Where multiple measures were available for the same construct, the choice of the marker was guided by the literature and model fit indices [i.e., Akaike Information Criterion (AIC) and Bayesian Information Criterion (BIC)]. Additional factors were considered confounders if associated with timing of ART and WAZ or LAZ in the bivariate analysis with *P* < 0.1. Hypothesized interactions with the primary exposure were included if model fit was improved based on AIC and BIC (Figure 1). Confounders identified through the bivariate analysis and interactions with splines were included in final models if *P* < 0.1.

To explore the association between timing of ART and WAZ and LAZ, an unadjusted model was fitted to estimate the crude association (model 1). Multivariable models were used to adjust the associations for confounders (model 2), and as LBW and child morbidity may be on the causal pathway between timing of ART exposure and child growth (Figure 1), we fitted two additional models to explore the effect of potential mediation by these factors (models 3–4).

To further assess the robustness of findings, we conducted sensitivity analyses including only CHEU with complete anthropometry data at all three visits. First, we described WAZ and LAZ scores at each visit, and second, we repeated all four mixed-effects linear regression models (models 1–4) in this group.

### Ethics Approvals

Ethical approval for the NEMAPP study was obtained from the Malawi National Health Sciences Research Committee (NHSRC, #1262), the US Centers for Disease Control and Prevention Global Health Associate Director for Science (#2014-054-7), and the University of Toronto Research Ethics Committee (#30448). Ethical approval for this study was obtained from University College London (UCL) Research Ethics Committee (Ref: 3715/004).

## Results

### Enrollment Characteristics

Overall 1,206 singleton CHEU and their mothers were enrolled in the study and 563 (46.7%) completed follow-up through 24 months of age. Among these mother–child pairs, 1,185 had timing of ART exposure available and were included in the growth analysis, with 61.4% (727/1,185) attending visit 1 and 46.7% (553/1,185) attending visit 2. All CHEU included in this analysis were HIV-free through 24 months of age. Enrollment took place at CHEU median age of 2 months [interquartile range (IQR): 2,4; range: 1–6], visit 1 at the median age of 12 months (IQR: 12,14; range: 9–20), and visit 2 at the median age of 24 months (IQR: 23,24; range: 18–33).

Median maternal age was 29 years (IQR: 24,33), mothers had a median 3 (IQR: 3,4) antenatal care visits and almost all mothers (99%) were on ART at enrollment; approximately 80% were receiving TDF + 3TC + EFV first-line ART regimen (Table 1A). Maternal viral load was detectable for 23% of mothers at study enrollment, and one-fifth were underweight. For CHEU, 50% of infants were female, 92% were exclusively breastfed, and 57% were registered with HIV care clinics (Table 1B). Almost half of CHEU (48%) were exposed to ART from conception, 40% from first or second trimester, and 12% from third trimester or postpartum. Approximately, 12% of CHEU had a LBW, almost all (99%) received nevirapine prophylaxis, and among CHEU aged ≥ 2 months (*n* = 1,173), nearly half (49%) were receiving co-trimoxazole prophylaxis at enrollment. Mothers on ART at conception were older, more likely to be multiparous, less likely to be from urban areas, and more likely to report being “a little bit sick” at ART initiation than those starting later; however, fewer of this group had detectable HIV viral load at study enrollment than mothers who started ART later (Table 1A). CHEU exposed to ART from conception received more days of nevirapine prophylaxis and were more likely to be enrolled in HIV care clinics than CHEU exposed to ART in the third trimester or postpartum (Table 1B).

**TABLE 1A:** Enrollment maternal and contextual characteristics.

	Cohort	Timing of antiretroviral therapy exposure				Total
		Conception	1*^st^*/2*^nd^* trimester	3*^rd^*trimester/PP	p-value	
**Maternal characteristics**	N = 1,185	n = 570	n = 475	n = 140		1,185
Age					< 0.001	1,182
<21 years	110 (9.3%)	23 (4.1%)	64 (13.5%)	23 (16.4%)		
21–34 years	860 (72.8%)	396 (69.8%)	357 (75.2%)	107 (76.4%)		
≥35 years	212 (17.9%)	148 (26.1%)	54 (11.4%)	10 (7.1%)		
Parity					< 0.001	1,185
Primiparous	173 (14.6%)	41 (7.2%)	112 (23.6%)	20 (14.3%)		
Multiparous	1,012 (85.4%)	529 (92.8%)	363 (76.4%)	120 (85.7%)		
Antenatal care visits					0.034	1,177
1–2 visits	203 (17.2%)	93 (16.4%)	74 (15.7%)	36 (26.1%)		
3–4 visits	803 (68.2%)	391 (68.8%)	323 (68.6%)	89 (64.5%)		
5+ visits	171 (14.5%)	84 (14.8%)	74 (15.7%)	13 (9.4%)		
HIV status at enrollment					< 0.001	1,184
Already known positive	1,136 (95.9%)	569 (100.0%)	475 (100.0%)	92 (65.7%)		
Newly diagnosed positive through study	48 (4.1%)	0 (0.0%)	0 (0.0%)	48 (34.3%)		
Health at ART initiation					< 0.001	1,116
No illness	910 (81.5%)	411 (73.7%)	420 (89.7%)	79 (87.8%)		
A little bit sick	159 (14.2%)	106 (19.0%)	42 (9.0%)	11 (12.2%)		
Very sick	47 (4.2%)	41 (7.3%)	6 (1.3%)	0 (0.0%)		
HIV viral load at enrollment					< 0.001	1,156
Detectable	261 (22.6%)	86 (15.5%)	99 (21.3%)	76 (55.9%)		
Undetectable	895 (77.4%)	470 (84.5%)	365 (78.7%)	60 (44.1%)		
Current ART use at enrollment					0.830	1,177
No, stopped ART	8 (0.7%)	3 (0.5%)	4 (0.8%)	1 (0.7%)		
Yes, on ART now	1,169 (99.3%)	561 (99.5%)	469 (99.2%)	139 (99.3%)		
Antiretrovirals missed in the past 30 days					0.087	1,111
0 days	883 (79.5%)	457 (82.0%)	363 (78.1%)	63 (70.8%)		
1 day	92 (8.3%)	44 (7.9%)	39 (8.4%)	9 (10.1%)		
≥2 days	136 (12.2%)	56 (10.1%)	63 (13.5%)	17 (19.1%)		
Maternal MUAC at enrollment					0.600	867
Normal	693 (79.9%)	320 (79.2%)	290 (81.5%)	83 (77.6%)		
Underweight	174 (20.1%)	84 (20.8%)	66 (18.5%)	24 (22.4%)		
**Contextual characteristics**						
Maternal spouse or partner					0.450	1,181
No	67 (5.7%)	32 (5.6%)	24 (5.1%)	11 (7.9%)		
Yes	1,114 (94.3%)	535 (94.4%)	450 (94.9%)	129 (92.1%)		
Maternal education					0.290	1,183
None	102 (8.6%)	56 (9.8%)	38 (8.0%)	8 (5.8%)		
Primary education	669 (56.6%)	331 (58.2%)	262 (55.2%)	76 (54.7%)		
Secondary education	391 (33.1%)	172 (30.2%)	165 (34.7%)	54 (38.8%)		
Post-secondary education	21 (1.8%)	10 (1.8%)	10 (2.1%)	1 (0.7%)		
Maternal employment					0.013	1,180
Unemployed	776 (65.8%)	350 (61.7%)	325 (68.6%)	101 (72.7%)		
Employed	404 (34.2%)	217 (38.3%)	149 (31.4%)	38 (27.3%)		
Geographical region					0.002	1,185
Blantyre (urban)	372 (31.4%)	151 (26.5%)	170 (35.8%)	51 (36.4%)		
Lilongwe (urban)	360 (30.4%)	167 (29.3%)	151 (31.8%)	42 (30.0%)		
North and Central (rural)	305 (25.7%)	170 (29.8%)	107 (22.5%)	28 (20.0%)		
South (rural)	148 (12.5%)	82 (14.4%)	47 (9.9%)	19 (13.6%)		
Travel time from home to clinic					0.270	1,173
<1 h	628 (53.5%)	311 (55.4%)	239 (50.5%)	78 (56.1%)		
1–2 h	448 (38.2%)	200 (35.7%)	199 (42.1%)	49 (35.3%)		
>2 h	97 (8.3%)	50 (8.9%)	35 (7.4%)	12 (8.6%)		

*Data presented are n (%); abbreviations: 1st, first trimester; 2nd, second trimester; 3rd, third trimester; PP, postpartum; ART, antiretroviral therapy.*

**TABLE 1B:** Enrollment child characteristics.

	Timing of antiretroviral therapy exposure					
	Cohort	Conception	1st/2nd trimester	3rd trimester/PP	*P*-value	Total
**Child characteristics**	*N* = 1,185	*n* = 570	*n* = 475	*n* = 140		
Age (months)	2.0 [2.0, 4.0]	2.0 [2.0, 4.0]	2.0 [2.0, 3.0]	2.0 [2.0, 4.0]	0.360	1,185
Sex					0.290	1,185
Male	597 (50.4%)	275 (48.2%)	245 (51.6%)	77 (55.0%)		
Female	588 (49.6%)	295 (51.8%)	230 (48.4%)	63 (45.0%)		
Born in health facility or clinic					0.380	1,180
No	54 (4.6%)	29 (5.1%)	17 (3.6%)	8 (5.8%)		
Yes	1,126 (95.4%)	537 (94.9%)	458 (96.4%)	131 (94.2%)		
Low birthweight					0.409	1,140
No	1,006 (88.2%)	486 (88.2%)	400 (87.3%)	120 (91.6%)		
Yes	134 (11.8%)	65 (11.8%)	58 (12.7%)	11 (8.4%)		
Breastfed in past 7 days					0.980	1,184
No breast milk	23 (1.9%)	11 (1.9%)	9 (1.9%)	3 (2.1%)		
Breast milk	1,161 (98.1%)	558 (98.1%)	466 (98.1%)	137 (97.9%)		
Sick or well at study enrollment					0.380	1,181
Sick	117 (9.9%)	62 (10.9%)	40 (8.4%)	15 (10.8%)		
Well	1,064 (90.1%)	506 (89.1%)	434 (91.6%)	124 (89.2%)		
History of infectious disease diagnosis					0.750	1,180
No	1,112 (94.2%)	536 (94.4%)	446 (94.5%)	130 (92.9%)		
Yes	68 (5.8%)	32 (5.6%)	26 (5.5%)	10 (7.1%)		
Number of sick visits to health facility/clinic					0.300	1,177
0 Times	936 (79.5%)	457 (80.9%)	373 (79.0%)	106 (75.7%)		
1 Time	188 (16.0%)	82 (14.5%)	82 (17.4%)	24 (17.1%)		
2+ Times	53 (4.5%)	26 (4.6%)	17 (3.6%)	10 (7.1%)		
Ever been admitted to hospital					0.303	1,181
No	1,125 (95.3%)	544 (95.9%)	451 (95.1%)	130 (92.9%)		
Yes	56 (4.7%)	23 (4.1%)	23 (4.9%)	10 (7.1%)		
Enrolled in HIV Care Clinic					<0.001	1,174
No	502 (42.8%)	216 (38.3%)	196 (41.6%)	90 (64.7%)		
Yes	672 (57.2%)	348 (61.7%)	275 (58.4%)	49 (35.3%)		
Received nevirapine prophylaxis					<0.001	1,114
No	10 (0.9%)	2 (0.4%)	1 (0.2%)	7 (6.9%)		
Yes	1,104 (99.1%)	548 (99.6%)	462 (99.8%)	94 (93.1%)		
Received co-trimoxazole prophylaxis*					<0.001	1,124
No	577 (51.3%)	254 (46.9%)	232 (50.8%)	91 (72.8%)		
Yes	547 (48.7%)	288 (53.1%)	225 (49.2%)	34 (27.2%)		
Weight-for-age Z-score**						
Enrollment (age 1–6 months)	–0.64 [–1.52, 0.17]	–0.52 [–1.38, 0.18]	–0.71 [–1.54, 0.10]	–0.58 [–1.53, 0.39]	0.581	1,165
Visit 1 (approx. 12 months)	–0.42 [–1.13, 0.33]	–0.44 [–1.15, 0.36]	–0.42 [–1.03, 0.33]	–0.13 [–1.13, 0.57]	0.522	710
Visit 2(approx. 24 months)	–0.82 [–1.43, –0.11]	–0.87 [–1.48, –0.11]	–0.73 [–1.29, –0.11]	–0.78 [–1.33, 0.31]	0.507	534
Length-for-age Z-score**						
Enrollment (age 1–6 months)	–2.10 [–3.32, –1.02]	–2.00 [–3.42, –1.02]	–2.13 [–3.22, –1.00]	–2.17 [–3.62, –1.17]	0.630	1,096
Visit 1 (approx. 12 months)	1.23 [–2.03, –0.39]	–1.29 [–2.33, –0.39]	–1.17 [–1.99, –0.31]	–1.23 [–1.99, –0.31]	0.662	690
Visit 2 (approx. 24 months)	–1.70 [–2.66, –0.95]	–1.74 [–2.76, –0.98]	–1.68 [–2.37, –0.98]	–1.78 [–2.66, –0.87]	0.695	535

Data presented are n (%) or median [IQR]; abbreviations: 1st, first trimester; 2nd, second trimester; 3rd, trimester; PP, postpartum. *Restricted to CHEU aged ≥ 2 months at study enrollment. **Enrollment took place at the median age of 2 months [interquartile range (IQR): 2, 4: range 1–6]; visit 1 at the median age of 12 months [IQR: 12, 14: range 9–20]; visit 2 at the median age of 24 months [IQR: 23, 24: range 18–33].

There were some differences in the enrollment characteristics of mother-child pairs who contributed to WAZ and/or LAZ mixed-effects models and those who did not (Supplementary Tables 1A,B). Mothers who were not included in models were more likely to be primiparous, had fewer antenatal care visits, were more likely to initiate ART in the third trimester or postpartum, and were more likely to present with a detectable HIV viral load than mothers who were included in models (Supplementary Table 1A). CHEU who were not included in models were more likely to be enrolled in HIV care clinics at study enrollment and were more likely to be from urban areas than CHEU who were included in models (Supplementary Table 1B).

### Child Anthropometry by Timing of ART Exposure and Age

**FIGURE 2:**
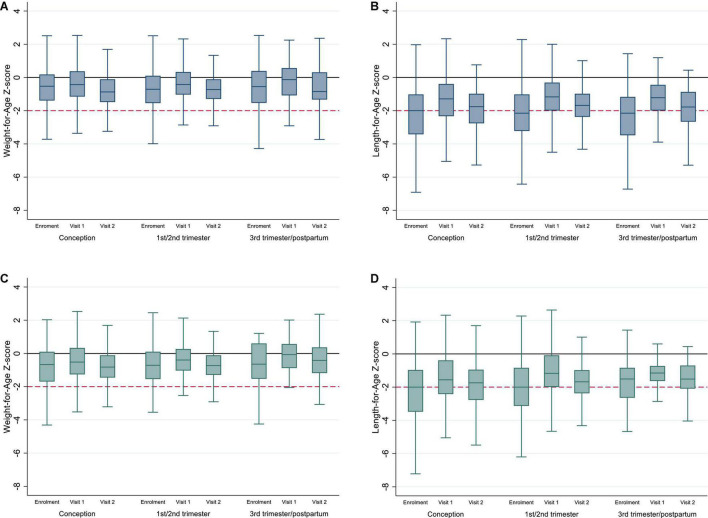
**(A,B)** Weight-for-Age and Length-for-Age Z-scores for whole cohort of CHEU by timing of ART exposure and study visit; **(C,D)** Weight-for-Age and Length-for-Age Z-scores for complete cases by timing of ART exposure and study visit. Y-line at 0 represents median WAZ and LAZ for the reference population, and y-line at -2 represents underweight **(A,C)** and stunting **(B,D)**. Outliers have been excluded from the box plots.

Anthropometry was available at enrollment, visit 1, and visit 2 for 98.3% (1,165/1,185), 97.7% (710/727), and 96.78% (535/553), respectively. Child anthropometry by timing of ART exposure and study visit are presented in Table 1B and Figure 2. Overall, median WAZ and LAZ were consistently below the reference median and demonstrated cohort-wide growth faltering after the ages of 12 and 13 months, respectively. Among CHEU with both WAZ and LAZ available, 21.3% were stunted at visit 1 (*n* = 687) increasing to 33.3% at visit 2 (*n* = 528) with no difference by timing of ART exposure; a smaller increase was observed in the proportion of CHEU underweight and stunted (4.5% at visit 1 and 7.6% at visit 2), and a slight decrease was observed in the proportion underweight only (2.6% at visit 1 and 2.1% at visit 2). Findings were similar in complete-cases (Supplementary Table 2 and Figure 2).

### Characteristics at Follow-Up Visits

Among 1,185 mother–child pairs, 67.7% (802/1,185) were included in WAZ or LAZ mixed-effects linear regression models; 796/802, 526/802, and 385/802 contributed at enrollment, visit 1, and visit 2, respectively. Overall, 5.5% (44/796) of CHEU had an infectious disease diagnosis in the 3 months before enrollment (or since birth); 58.9% (310/526) between enrollment and visit 1 and 39.7% (153/385) between visit 1 and 2. At visit 1, 90.5% (476/526) of CHEU were breastfed in the past 7 days, which declined to 6.0% (23/385) by visit 2. CHEU receipt of co-trimoxazole prophylaxis increased from 44.4% (346/779) at enrollment to 98.3% (517/526) at visit 1 and declined to 83.3% (320/384) at visit 2; missing data in the denominator for enrollment and visit 2 represent observations contributing to LAZ but not WAZ models. The majority of CHEU (>90%) were reported to be “well” at visits 1 (483/526) and 2 (372/385), and the proportion ever hospitalized increased over time [5.1% (41/796), 10.7% (56/526), and 16.6% (64/385) at enrollment, visit 1, and visit 2, respectively].

### Multivariable Mixed-Effects Regression up to 24 Months of Age

**FIGURE 3:**
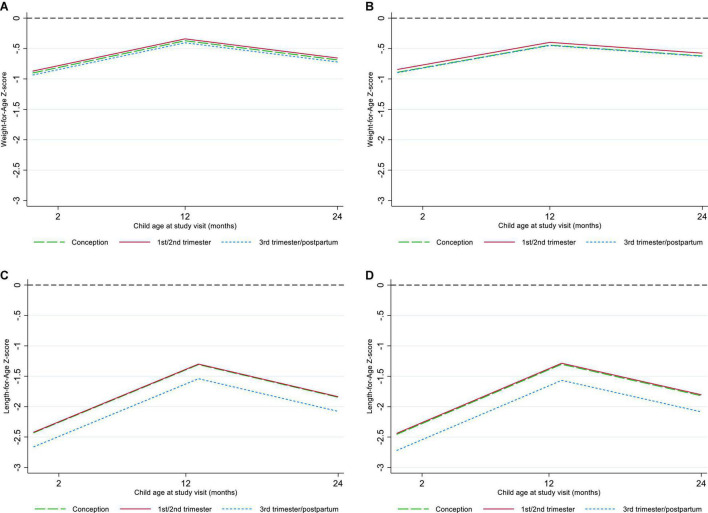
Marginal plots of Weight-for-Age and Length-for-Age by timing of antiretroviral therapy exposure. **(A)** Crude Weight-for-Age, **(B)** confounder-adjusted Weight-for-Age, **(C)** crude Length-for-Age, **(D)** confounder-adjusted Length-for-Age.

Overall, 788 and 780 CHEU with data across confounders and potential mediators contributed to the WAZ and LAZ mixed-effects models, respectively (Table 2). Adjusted for maternal, child, and contextual confounders (model 2), there was no statistical evidence of differences in mean WAZ or in WAZ rate of change through 24 months of age (all interactions *P* > 0.1) in CHEU exposed to ART from conception (WAZ: β = -0.006 [95% CI: -0.255, 0.267]) and first or second trimester (WAZ: β = 0.052 [-0.208, 0.312]) compared with those exposed in the third trimester or postpartum. In LAZ models adjusting for maternal, child, and contextual confounders (model 2), comparing CHEU exposed to ART from third trimester or postpartum with CHEU exposed to ART from conception (LAZ: β = 0.265 [-0.092, 0.622]) and first or second trimester (LAZ: β = 0.281 [-0.074, 0.637]) indicated no statistical evidence of differences in mean LAZ or in LAZ rate of change (all interactions *P* > 0.1) through 24 months (Figure 3).

**TABLE 2:** Differences in children HIV-exposed and uninfected longitudinal growth by timing of ART exposure by mixed-effects linear regression analysis.

	Weight-for-Age (*n* = 788)	Length-for-Age (*n* = 780)		
	Mean difference (95% CI)	*P*-value	Mean difference (95% CI)	*P*-value
**Model 1: Crude regression model**				
Conception	0.066 (–0.190, 0.321)	0.615	0.232 (–0.112, 0.576)	0.187
1st/2nd trimester	0.095 (–0.164, 0.355)	0.470	0.241 (–0.108, 0.590)	0.176
3rd trimester/postpartum	Ref		Ref	
Model 2: Confounder-adjusted*				
Conception	0.006 (–0.255, 0.267)	0.963	0.265 (–0.092, 0.622)	0.146
1st/2nd trimester	0.052 (–0.208, 0.312)	0.696	0.281 (–0.074, 0.637)	0.121
3rd trimester/postpartum	Ref		Ref	
Model 3: Confounder-adjusted**				
Conception	–0.013 (–0.272, 0.246)	0.922	0.252 (–0.104, 0.607)	0.166
1st/2nd trimester	0.028 (–0.231, 0.287)	0.832	0.247 (–0.107, 0.602)	0.172
3rd trimester/postpartum	Ref		Ref	
Model 4: Confounder-adjusted***				
Conception	0.006 (–0.238, 0.251)	0.959	0.287 (–0.059, 0.634)	0.104
1st/2nd trimester	0.034 (–0.210, 0.278)	0.785	0.266 (–0.079, 0.611)	0.131
3rd trimester/postpartum	Ref		Ref	

*1st, first trimester; 2nd, second trimester; 3rd, third trimester; LBW, low birthweight.*

*Model 2 was adjusted for a priori confounders (infant sex and age, breastfeeding in the past 7 days, maternal age, parity, maternal employment, maternal HIV viral load, maternal self-reported health at ART initiation, maternal MUAC, and geographical region) as well as potential confounders identified in bivariate analyses (weight-for-age models were adjusted for child receipt of co-trimoxazole prophylaxis with P < 0.1 in fitted model; no additional confounders were fitted in length-for-age models).

**Model 3 was adjusted for all confounders in model 2 and markers for child morbidity (child health, history of infectious disease diagnosis, and child ever hospitalized).

***Model 4 was adjusted for all confounders in model 3 and infant LBW.

For both WAZ and LAZ (model 2), evaluating the hypothesized interactions between timing of ART exposure and child age, breastfeeding, or maternal viral load at study enrollment showed no differences (*P* > 0.1) or model fit improvement. Adjustment for CHEU morbidity (model 3) and LBW (model 4) demonstrated no evidence of mediation (*P* > 0.1). In complete cases (model 2), statistical inferences on the effect of timing of ART exposure on WAZ and LAZ and potential mediation (models 3–4) remained the same; there was a trend toward lower WAZ and LAZ among CHEU exposed to ART from conception compared with CHEU exposed later, but this did not reach statistical significance (Supplementary Table 3 and Figure 1).

## Discussion

In the context of high antenatal ART coverage and breastfeeding uptake in Malawi, we found CHEU growth was suboptimal, with linear growth faltering being more common than underweight at approximately 24 months of age. Our study observed no differences in the early growth trajectories of CHEU by timing of maternal ART initiation through 24 months of age.

Our data highlight that CHEU repeatedly presented with lower WAZ and LAZ than the reference population, with some evidence of early catch-up growth from enrollment to visit 1, although not prolonged as z-scores declined by visit 2. Our findings approximate those of previous Eastern and Southern African studies (10–15, 17, 21), including a large cohort of South African CHEU, where ART coverage in pregnancy was estimated at 96–97% (2013–2016) (37) and CHEU presented with negative LAZ between the ages of 6 weeks and 12 months, with some recovery between 6 weeks and 3 months of age (10). Consistent with our findings, several studies [conducted when antenatal ART coverage was estimated at 85–90% in Eastern and Southern Africa (37)] have reported a decline in WAZ or LAZ in CHEU starting as early as the age of 3 months and often between the ages of 6 and 9 months, around the time of introduction to complementary foods (10, 11, 17, 20). In our study, the decline in WAZ and LAZ after the ages of 12 and 13 months reflected cohort-wide growth faltering.

Given the mounting evidence that CHEU experience a higher risk of poor growth than CHU, there are efforts to explore potential mechanisms and contributors, including any potential role of duration of ART exposure. In our study, 88% of CHEU were exposed to ART from conception or first or second trimester and 98% *via* breast milk, comparable with population-level estimates (38). Reassuringly, our data present strong evidence of no difference in WAZ or LAZ by timing of ART exposure, similar to other findings in Eastern and Southern Africa (20, 21, 23). In a prospective cohort of South African and Zambian CHEU (2017–2018), exposure from conception or later during gestation was not associated with any differences in WAZ, LAZ, or WLZ at 6–10 weeks or 6 months, whereas a Malawian prospective cohort (2014–2016) observed no difference in mean weight or height in CHEU exposed to ART up to the age of 18 months (*via* breast milk) compared with CHU (20). Other studies have observed differences in CHEU growth by timing of ART exposure (17, 19, 22), including a retrospective analysis of Ethiopian CHEU in which infants exposed from conception presented with lower rate of change in length during the first 3 months of life and doubled odds of stunting up to the age of 12 months than infants exposed late in pregnancy (19). Despite these mixed findings, ART initiated before conception and continued throughout pregnancy has a crucial role in preventing vertical transmission of HIV (42) and, for those adhering to treatment, is also associated with low rates of postnatal transmission and improved maternal health (1, 36).

NRTIs have been associated with mitochondrial toxicity in people living with HIV (43, 44), are known to cross the placental barrier, and may be secreted into breast milk (45). Some studies have reported a negative effect of *in utero* exposure to TDF on growth because of its association with lower LAZ, WAZ, and bone mineral content (25–27, 46), though findings are inconsistent (23, 24, 47). Proposed mechanisms by which fetal TDF exposure may result in growth deficiencies include NRTI toxicity on mitochondrial DNA and damage to nuclear DNA of hematopoietic stem cells (48, 49). Data from children living with HIV suggest that TDF regimens may cause abnormal growth through proximal renal tubular dysfunction with hyperphosphaturia or disruption of vitamin D metabolism (50). In our study, we were unable to assess the effects of different antiretroviral drugs due to homogeneity in ART regimens. Additional research and pharmacovigilance are needed to assess the long-term safety of TDF and other antiretrovirals (51), especially as the proportion of CHEU exposed to ART from conception increases.

Studies from both high-income and low-income countries have observed a potentially causal association between ART exposure from conception and increased risk of adverse birth outcomes, including preterm birth, small-for-gestational-age (SGA), and LBW (28, 30, 33, 35). In a previous meta-analysis, WLHIV who conceived on ART had 30% greater risk of a LBW infant than women who started ART during pregnancy (28), and in a more recent study from Botswana, treatment with TDF + 3TC + EFV less than 1 year before conception increased the risk of preterm delivery by 33% compared with later initiation (33). Additionally, investigators reported that LBW and very LBW infants were most common among women who started TDF + 3TC + EFV between 0 and 7 weeks gestation, but this did not reach statistical significance (33).

In our study, 48% of CHEU were exposed to ART from conception and 12% had LBW overall, similar to national estimates of LBW (38), but may be underestimated, given this was a survival cohort. Although we hypothesized that the effects of timing of ART exposure on CHEU growth may be partially mediated through LBW, we saw negligible differences after adjustment for LBW. Despite this, prematurity, SGA, and LBW are consistently associated with poor postnatal growth in low- and middle-income countries (3, 52). For instance, in a recent South African prospective study, compared with CHU-born SGA, CHEU-born SGA with suboptimal fetal growth presented with significantly worse growth trajectories through the first 12 months of life (10). This suggests that restricted *in utero* growth may be a developmental origin of CHEU suboptimal growth (53, 54) and demonstrates CHEU risk of poor growth may be compounded by their earlier risk of adverse birth outcomes.

While our study observed no differences in CHEU growth by timing of ART exposure, these findings must be contextualized within the Malawian setting with high levels of malnutrition and poverty (39). As such, it is possible that the distribution and burden of well-established universal risk factors for poor growth were more prevalent in this cohort of CHEU, which may have masked small but significant differences in the effect of timing of ART exposure on growth. Nonetheless, other potential predictors of poor growth in this cohort may include maternal undernutrition and breastfeeding. In our study, 20% of mothers overall were undernourished and 98% of CHEU were breastfed in the past 7 days at study enrollment. Although breastfeeding prevalence was high at enrollment, maternal nutritional deficiencies may have reduced quantity or quality of their breast milk. At approximately 24 months of age, we found only 6% of mothers reported breastfeeding, which may be a reflection of HIV guidelines: the WHO recommends mothers breastfeed to Up to 24 months of age or longer, while the Malawi Ministry of Health recommends cessation at the age of 22 months so that a final HIV test can be carried out at 24 months. Unsurprisingly, breastfeeding in this cohort was lower than population-level estimates of 16% among children aged 24–36 months (38) and aligned with the decline in WAZ and LAZ observed after 12 and 13 months of age, respectively. Further investigation is needed to understand the effects of ART exposure through breast milk and to identify the optimal durations of breastfeeding for CHEU in resource-limited settings such as Malawi.

Our study has a few limitations as follows: mother-child pairs were enrolled at under-5 clinics, which may have missed women with pregnancy complications, severely ill neonates, or infants not attending routine visits. As most measures collected were self-reported by mothers, this carries a risk of recall bias, although maternal timing of ART initiation was validated using ART Patient Cards, and child anthropometry was recorded at clinics and was available for >95% of CHEU that attended each visit. Data collected on timing of ART initiation did not include precise dates, or disaggregation by first versus second trimester, and so exact timing could not be explored in more detail. While we were able to adjust for maternal viral load at study enrollment, as a proxy for viral load during pregnancy, no measures on maternal HIV disease stage or CD4 cell count at ART initiation or during pregnancy were available. As a result, there may be uncontrolled confounding in our models, particularly among mothers who initiated ART prior to conception; however, most mothers reported feeling “well” at ART initiation, suggesting this may have had minimal confounding effect. Women initiating ART in the third trimester/postpartum may have differed from those starting ART earlier in pregnancy in ways not fully captured by the data available, which could also have been relevant for child growth, resulting in residual confounding. Additionally, we were unable to assess potential mediation by preterm birth, which we hypothesized may be on the causal pathway, although we believe that adjustment for LBW partially captured preterm births in this cohort. A large proportion of the mother-child pairs did not contribute to mixed-effects models, and differences between those who were and were not included in models were reported and complete-case analyses were conducted. Growth deficiencies may have been under- or overestimated as the WHO growth reference we used was not based on a Malawian population (41), and data on child age were only available in whole months; this is not expected to have impacted overall growth trajectories and comparisons by timing of ART exposure. Without a control group of CHU, we were unable to explore the effect of HIV or fully explore the effect of ART exposure on longitudinal WAZ and LAZ.

Our data highlighted that CHEU exposure to ART from conception compared with later in pregnancy or postpartum was not associated with decreased WAZ or LAZ in CHEU during the first 2 years of life, supporting current WHO guidelines on maternal lifelong ART and breastfeeding. While programmatic efforts to improve CHEU health outcomes in Malawi include careful monitoring until a final negative HIV test result, due to ongoing HIV exposure through breastfeeding, growth trajectories from our data suggest CHEU and their families may need monitoring and support through and beyond the first 2 years of life.

## Data Availability Statement

The data analyzed in this study is subject to the following licenses/restrictions: the Malawi Ministry of Health prefer that data are not shared publicly and ethics and governance in place for the study does not permit public availability of the data as they consist of detailed individual-level patient data. Requests to access these datasets should be directed to BT, beth@nyanja-health.com.

## Ethics Statement

The studies involving human participants were reviewed and approved by Malawi National Health Sciences Research Committee (NHSRC, #1262), the US Centers for Disease Control and Prevention Global Health Associate Director for Science (#2014-054-7), the University of Toronto Research Ethics Committee (#30448), and University College London (UCL) Research Ethics Committee (Ref: 3715/004). Written informed consent to participate in this study was provided by the participants’ legal guardian/next of kin.

## Author Contributions

GT, CT, HB, and SC conceived the manuscript. GT conducted the analysis and drafted the manuscript. CT, HB, and SC supervised the work of GT. SC provided the statistical support. All authors contributed to the article and approved the submitted version.

## Author Disclaimer

The views expressed are those of the author(s) and not necessarily those of the NHS, the NIHR, or the Department of Health.

## Conflict of Interest

The authors declare that the research was conducted in the absence of any commercial or financial relationships that could be construed as a potential conflict of interest.

## Publisher’s Note

All claims expressed in this article are solely those of the authors and do not necessarily represent those of their affiliated organizations, or those of the publisher, the editors and the reviewers. Any product that may be evaluated in this article, or claim that may be made by its manufacturer, is not guaranteed or endorsed by the publisher.
