# Supplementary material for: No Difference in Growth Outcomes up to 24 Months of Age by Duration of Exposure to Maternal Antiretroviral Therapy Among Children Who Are HIV-Exposed and Uninfected in Malawi
Source: Front Pediatr. 2022 Jun 20;10:882468. doi: 10.3389/fped.2022.882468 (PMC9251312; doi:10.3389/fped.2022.882468)
Supplement: Supplementary file 1 [file Data_Sheet_1.docx]

| **Table S1a.** Enrolment maternal and contextual characteristics by mother-child pairs who did and did not contribute to mixed effects models. | | | | | |
| --- | --- | --- | --- | --- | --- |
|  | **Total** | **MCPs not included in mixed effects models** | **MCPs included in mixed effects models** | **p-value** | **Total** |
| **Maternal characteristics** | N=1,185 | N=383 | N=802 |  | 1,185 |
| Age |  |  |  | 0.520 | 1,182 |
| <21 years | 110 (9.3%) | 31 (8.2%) | 79 (9.9%) |  |  |
| 21-34 years | 860 (72.8%) | 284 (74.7%) | 576 (71.8%) |  |  |
| ≥35 years | 212 (17.9%) | 65 (17.1%) | 147 (18.3%) |  |  |
| Parity |  |  |  | 0.220 | 1,185 |
| Primiparous | 173 (14.6%) | 49 (12.8%) | 124 (15.5%) |  |  |
| Multiparous | 1,012 (85.4%) | 334 (87.2%) | 678 (84.5%) |  |  |
| Antenatal care visits |  |  |  | 0.240 | 1,177 |
| 1-2 visits | 203 (17.2%) | 74 (19.7%) | 129 (16.1%) |  |  |
| 3-4 visits | 803 (68.2%) | 252 (67.2%) | 551 (68.7%) |  |  |
| ≥5 visits | 171 (14.5%) | 49 (13.1%) | 122 (15.2%) |  |  |
| HIV status at enrolment |  |  |  | 0.003 | 1,184 |
| Already known positive | 1,136 (95.9%) | 358 (93.5%) | 778 (97.1%) |  |  |
| Newly diagnosed positive through study | 48 (4.1%) | 25 (6.5%) | 23 (2.9%) |  |  |
| Health at ART initiation |  |  |  | 0.200 | 1,116 |
| No illness | 910 (81.5%) | 281 (79.8%) | 629 (82.3%) |  |  |
| A little bit sick | 159 (14.2%) | 59 (16.8%) | 100 (13.1%) |  |  |
| Very sick | 47 (4.2%) | 12 (3.4%) | 35 (4.6%) |  |  |
| Timing of ART initiation |  |  |  | 0.410 | 1,185 |
| Conception | 570 (48.1%) | 192 (50.1%) | 378 (47.1%) |  |  |
| 1st/2nd trimester | 475 (40.1%) | 143 (37.3%) | 332 (41.4%) |  |  |
| 3rd trimester/postpartum | 140 (11.8%) | 48 (12.5%) | 92 (11.5%) |  |  |
| HIV viral load at enrolment |  |  |  | 0.280 | 1,156 |
| Detectable | 261 (22.6%) | 87 (24.6%) | 174 (21.7%) |  |  |
| Undetectable | 895 (77.4%) | 267 (75.4%) | 628 (78.3%) |  |  |
| Current ART use at enrolment |  |  |  | 0.670 | 1,177 |
| No, stopped ART | 8 (0.7%) | 2 (0.5%) | 6 (0.8%) |  |  |
| Yes, on ART now | 1,169 (99.3%) | 375 (99.5%) | 794 (99.3%) |  |  |
| Antiretrovirals missed in the past 30 days |  |  |  | 0.760 | 1,111 |
| 0 days | 883 (79.5%) | 282 (80.8%) | 601 (78.9%) |  |  |
| 1 day | 92 (8.3%) | 27 (7.7%) | 65 (8.5%) |  |  |
| 2 or more days | 136 (12.2%) | 40 (11.5%) | 96 (12.6%) |  |  |
| **Contextual factors** |  |  |  |  |  |
| Maternal spouse or partner |  |  |  | 0.350 | 1,181 |
| No | 67 (5.7%) | 18 (4.7%) | 49 (6.1%) |  |  |
| Yes | 1,114 (94.3%) | 361 (95.3%) | 753 (93.9%) |  |  |
| Maternal education |  |  |  | 0.083 | 1,183 |
| None | 102 (8.6%) | 32 (8.4%) | 70 (8.7%) |  |  |
| Primary education | 669 (56.6%) | 235 (61.7%) | 434 (54.1%) |  |  |
| Secondary education | 391 (33.1%) | 109 (28.6%) | 282 (35.2%) |  |  |
| Post-secondary education | 21 (1.8%) | 5 (1.3%) | 16 (2.0%) |  |  |
| Maternal employment |  |  |  | <0.001 | 1,180 |
| Unemployed | 776 (65.8%) | 275 (72.8%) | 501 (62.5%) |  |  |
| Employed | 404 (34.2%) | 103 (27.2%) | 301 (37.5%) |  |  |
| Geographical region |  |  |  | <0.001 | 1,185 |
| Blantyre Urban | 372 (31.4%) | 20 (5.2%) | 352 (43.9%) |  |  |
| Lilongwe Urban | 360 (30.4%) | 247 (64.5%) | 113 (14.1%) |  |  |
| North and Central rural | 305 (25.7%) | 115 (30.0%) | 190 (23.7%) |  |  |
| South rural | 148 (12.5%) | 1 (0.3%) | 147 (18.3%) |  |  |
| Travel time from clinic to home in hours |  |  |  | 0.004 | 1,173 |
| <1 hour | 628 (53.5%) | 173 (46.5%) | 455 (56.8%) |  |  |
| 1-2 hours | 448 (38.2%) | 162 (43.5%) | 286 (35.7%) |  |  |
| >2 hours | 97 (8.3%) | 37 (9.9%) | 60 (7.5%) |  |  |

Data are n (%); Abbreviations: MCPs: mother-child pairs; 24mo: 24 months of age; FUP: follow-up; ART: antiretroviral therapy.

| **Table S1b.** Enrolment child characteristics by mother-child pairs who did and did not contribute to mixed effects models. | | | | |  |
| --- | --- | --- | --- | --- | --- |
|  | **Overall cohort** | **MCPs not included in mixed effects models** | **MCPs included in mixed effects models** | **p-value** | **Total** |
| **Child characteristics** | N=1,185 | N=383 | N=802 |  | 1,185 |
| Age | 2.0 [2.0, 4.0] | 3.0 [2.0, 4.0] | 2.0 [2.0, 3.0] | 0.007 | 1,185 |
| Sex |  |  |  | 0.450 | 1,185 |
| Male | 597 (50.4%) | 199 (52.0%) | 398 (49.6%) |  |  |
| Female | 588 (49.6%) | 184 (48.0%) | 404 (50.4%) |  |  |
| Child born in health facility or clinic |  |  |  | <0.001 | 1,180 |
| No | 54 (4.6%) | 31 (8.1%) | 23 (2.9%) |  |  |
| Yes | 1,126 (95.4%) | 351 (91.9%) | 775 (97.1%) |  |  |
| Low birthweight |  |  |  | 0.670 | 1,141 |
| Normal birthweight | 1,006 (88.2%) | 301 (88.8%) | 705 (87.9%) |  |  |
| low birthweight | 135 (11.8%) | 38 (11.2%) | 97 (12.1%) |  |  |
| Breastfed in the past 7 days |  |  |  | 0.480 | 1,184 |
| No breastmilk | 23 (1.9%) | 9 (2.4%) | 14 (1.7%) |  |  |
| Breastmilk | 1,161 (98.1%) | 373 (97.6%) | 788 (98.3%) |  |  |
| History of infectious disease diagnosis |  |  |  | 0.550 | 1,180 |
| No | 1,112 (94.2%) | 354 (93.7%) | 758 (94.5%) |  |  |
| Yes | 68 (5.8%) | 24 (6.3%) | 44 (5.5%) |  |  |
| Number of sick visits to health facility or clinic |  |  |  | 0.940 | 1,177 |
| 0 times | 936 (79.5%) | 298 (79.5%) | 638 (79.6%) |  |  |
| 1 time | 188 (16.0%) | 59 (15.7%) | 129 (16.1%) |  |  |
| 2+ times | 53 (4.5%) | 18 (4.8%) | 35 (4.4%) |  |  |
| Ever been admitted to hospital |  |  |  | 0.380 | 1,181 |
| No | 1,125 (95.3%) | 364 (96.0%) | 761 (94.9%) |  |  |
| Yes | 56 (4.7%) | 15 (4.0%) | 41 (5.1%) |  |  |
| Enrolled in HIV Care Clinic |  |  |  | <0.001 | 1,174 |
| No | 502 (42.8%) | 126 (33.6%) | 376 (47.1%) |  |  |
| Yes | 672 (57.2%) | 249 (66.4%) | 423 (52.9%) |  |  |
| Received nevirapine prophylaxis |  |  |  | 0.900 | 1,114 |
| No | 10 (0.9%) | 3 (0.8%) | 7 (0.9%) |  |  |
| Yes | 1,104 (99.1%) | 351 (99.2%) | 753 (99.1%) |  |  |
| Received co-trimoxazole prophylaxis* |  |  |  | <0.001 | 1,124 |
| No | 577 (51.3%) | 145 (42.2%) | 432 (55.4%) |  |  |
| Yes | 547 (48.7%) | 199 (57.9%) | 348 (44.6%) |  |  |
| Weight-for-Age Z-score** |  |  |  |  |  |
| Enrolment | -0.64 [-1.52, 0.17] | -0.48 [-1.42, 0.25] | -0.71 [-1.54, 0.10] | 0.013 | 1,165 |
| Visit 1 | -0.42 [-1.13, 0.33] | -0.42 [-1.03, 0.37] | -0.42 [-1.13, 0.33] | 0.799 | 710 |
| Visit 2 | -0.82 [-1.43, -0.11] | -0.73 [-1.57, -0.11] | -0.83 [-1.39, -0.11] | 0.690 | 534 |
| Length-for-Age Z-score** |  |  |  |  |  |
| Enrolment | -2.10 [-3.32, -1.02] | -2.15 [-3.32, -1.02] | -2.00 [-3.32, -1.02] | 0.786 | 1,096 |
| Visit 1 | 1.23 [-2.03, -0.39] | -1.17 [-1.94, -0.38] | -1.29 [-2.33, -0.39] | 0.297 | 690 |
| Visit 2 | -1.70 [-2.66, -0.95] | -1.51 [-2.66, -0.93] | -1.78 [-2.66, -0.98] | 0.708 | 535 |

Data presented are n (%) or median [IQR]; Abbreviations: MCPs: mother-child pairs; 24mo: 24 months of age; FUP: follow-up. *Restricted to CHEU aged ≥ 2 months at study enrolment. **Enrolment took place at median age 2 months [interquartile range (IQR): 2, 4: range 1-6); visit 1 at median age 12 months [IQR: 12, 14: range 9-20]; visit 2 at median age 24 months [IQR: 23, 24: range 18-33].

| **Table S2.** Child anthropometry by timing of ART exposure and age in complete-cases. | | | | | |
| --- | --- | --- | --- | --- | --- |
| **Weight-for-Age*** | **Total (n=446)** | **Conception (n=229)** | **1^st^/2^nd^ trimester (n=181)** | **3^rd^ trimester/PP (n=36)** | **p-value** |
| Enrolment | -0.67 [-1.55, 0.10] | -0.67 [-1.69, 0.10] | -0.71 [-1.53, 0.10] | -0.65 [-1.53, 0.60] | 0.667 |
| Visit 1 | -0.43 [-1.13, 0.33] | -0.52 [-1.26, 0.33] | -0.42 [-1.03, 0.27] | -0.07 [-0.88, 0.57] | 0.156 |
| Visit 2 | -0.74 [-1.35, -0.06] | -0.83 [-1.45, -0.11] | -0.73 [-1.29, -0.11] | -0.42 [-1.18, 0.37] | 0.507 |
| **Length-for-Age*** | **Total (n=411)** | **Conception (n=201)** | **1^st^/2^nd^ trimester (n=177)** | **3^rd^ trimester/PP (n=33)** | **p-value** |
| Enrolment | -2.00 [-3.26, -0.91] | -2.00 [-3.48, -0.97] | -2.00 [-3.13, -0.84] | -1.51 [-2.64, -0.84] | 0.367 |
| Visit 1 | -1.21 [-1.99, -0.31] | -1.56 [-2.42, -0.39] | -1.17 [-1.99, -0.09] | -1.15 [-1.63, -0.73] | 0.152 |
| Visit 2 | -1.68 [-2.66, -0.85] | -1.74 [-2.77, -0.95] | -1.68 [-2.37, -0.98] | -1.68 [-2.09, -0.70] | 0.601 |

Abbreviations: 1^st^: first trimester; 2^nd^: second trimester; 3^rd^: third trimester.

*Enrolment took place at median age 2 months [interquartile range (IQR): 2, 4: range 1-6); visit 1 at median age 12 months [IQR: 12, 14: range 9-20]; visit 2 at median age 24 months [IQR: 23, 24: range 18-33].

| **Table S3. Differences in CHEU longitudinal growth by timing of ART exposure in complete-cases.** | | | | |
| --- | --- | --- | --- | --- |
|  | **Weight-for-Age (n=336)** | | **Length-for-Age (n=310)** | |
| **Model 1: Crude regression** | **Mean difference (95% CI)** | **p-value** | **Mean difference (95% CI)** | **p-value** |
| Conception | -0.153 (-0.510, 0.204) | 0.400 | -0.247 (-0.713, 0.220) | 0.300 |
| 1st/2nd trimester | 0.004 (-0.358, 0.366) | 0.983 | -0.047 (-0.515, 0.422) | 0.845 |
| 3rd trimester/postpartum | Ref |  | Ref |  |
| **Model 2: Confounder-adjusted *** | | | |  |
| Conception | -0.070 (-0.436, 0.295) | 0.706 | -0.091 (-0.564, 0.382) | 0.706 |
| 1st/2nd trimester | 0.072 (-0.297, 0.442) | 0.700 | 0.154 (-0.323, 0.631) | 0.526 |
| 3rd trimester/postpartum | Ref |  | Ref |  |
| **Model 3: Confounder-adjusted **** | | | |  |
| Conception | -0.084 (-0.449, 0.281) | 0.652 | -0.095 (-0.569, 0.379) | 0.696 |
| 1st/2nd trimester | 0.060 (-0.309, 0.428) | 0.752 | 0.130 (-0.349, 0.609) | 0.595 |
| 3rd trimester/postpartum | Ref |  | Ref |  |
| **Model 4: Confounder-adjusted ***** | | | |  |
| Conception | -0.005 (-0.342, 0.332) | 0.978 | -0.025 (-0.498, 0.435) | 0.915 |
| 1st/2nd trimester | 0.084 (-0.256, 0.424) | 0.628 | 0.127 (-0.337, 0.590) | 0.593 |
| 3rd trimester/postpartum | Ref |  | Ref |  |

Abbreviations: 1^st^: first trimester; 2^nd^: second trimester; 3^rd^: third trimester; LBW: low birthweight.

*Model 2 was adjusted for *a priori* confounders (infant sex and age, breastfeeding in the past 7 days, maternal age, parity, maternal employment, maternal HIV viral load, maternal self-reported health at ART initiation, maternal MUAC, and geographical region) as well as potential confounders identified in bivariate analyses (Weight-for-Age models were adjusted for child receipt of co-trimoxazole prophylaxis with P<0.1 in fitted model; no additional confounders were fitted in Length-for-Age models). **Model 3 was adjusted for all confounders in model 2 and markers for child morbidity (child health, history of infectious disease diagnosis, child ever hospitalized) ***Model 4 was adjusted for all confounders in model 3 and infant LBW.
