# Supplementary figures and images for: No Difference in Growth Outcomes up to 24 Months of Age by Duration of Exposure to Maternal Antiretroviral Therapy Among Children Who Are HIV-Exposed and Uninfected in Malawi
Source: Front Pediatr. 2022 Jun 20;10:882468. doi: 10.3389/fped.2022.882468 (PMC9251312; doi:10.3389/fped.2022.882468)

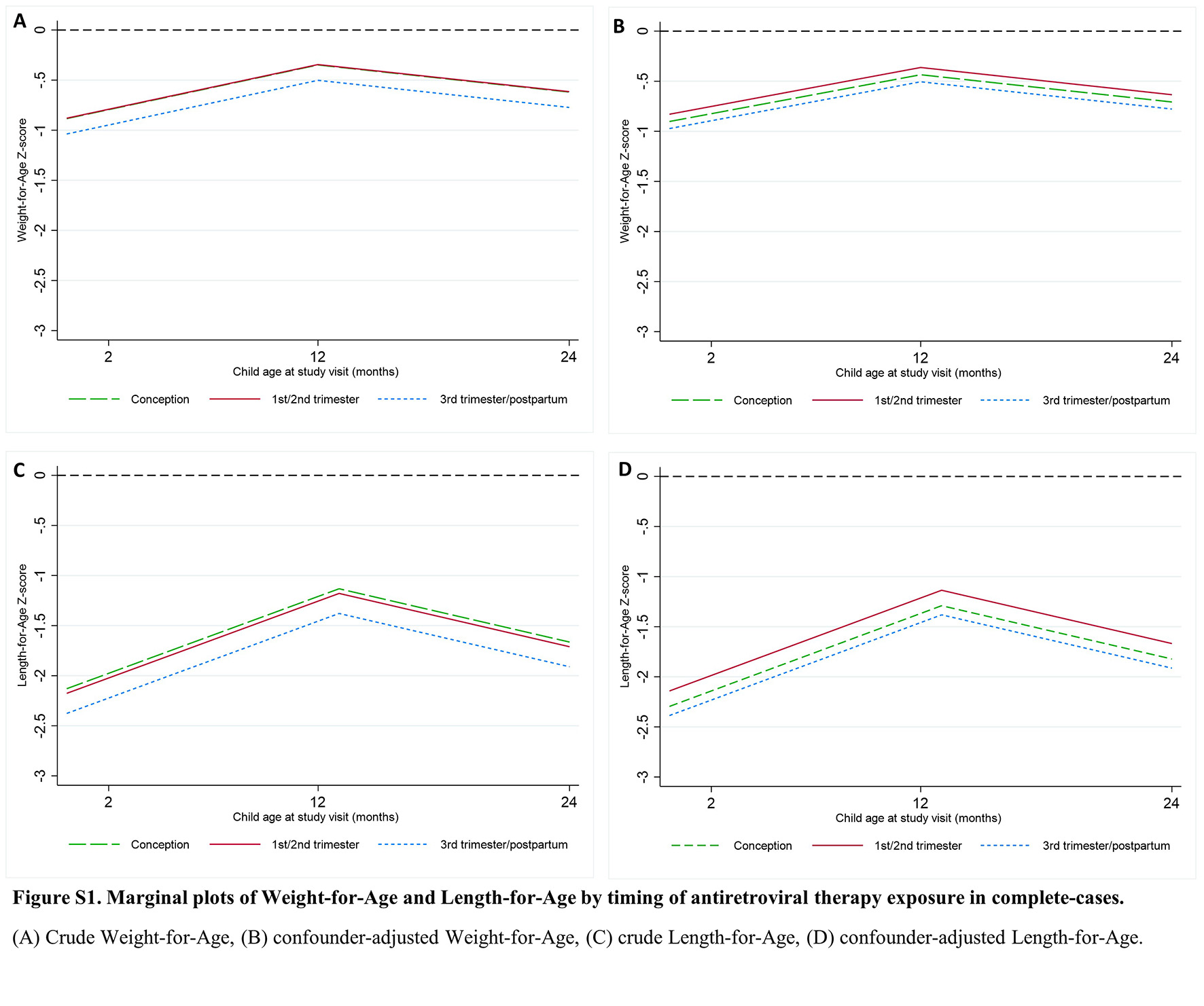

Supplement: Supplementary file 2 [file Image_1.jpeg]
